# Surveillance of avian malaria and related haemoparasites in common terns (*Sterna hirundo*) on the Atlantic coast of South America

**DOI:** 10.1017/S0031182023000185

**Published:** 2023-05

**Authors:** Sofía Capasso, Yvonne R. Schumm, Petra Quillfeldt, Claire Bonsergent, Laurence Malandrin, Eliana Lorenti, Bruno Fusaro, Guillermo Panisse, Melina Lunardelli, Gabriel Castresana, Julia I. Diaz

**Affiliations:** 1 Centro de Estudios Parasitológicos y de Vectores (CEPAVE), FCNyM, UNLP, CONICET, Boulevard 120 s/n e/61 y 62, 1900 La Plata, Argentina; 2 Department of Animal Ecology & Systematics, Justus Liebig University, Heinrich-Buff-Ring 26-32, IFZD-35392 Giessen, Germany; 3 Oniris, INRAE, BIOEPAR, 44300, Nantes, France; 4 Departamento de Ecofisiología y Ecotoxicología, Instituto Antártico Argentino (DNA), 25 de Mayo 1143, San Martín, Buenos Aires, Argentina; 5 Reserva Natural Bahía Samborombón, Dirección de Áreas Protegidas, Ministerio de Ambiente, Buenos Aires, Argentina

**Keywords:** Argentine Atlantic coast, avian blood parasites, Charadriiformes, cytochrome b gene, migratory birds, *Plasmodium*

## Abstract

Haemosporidia (Apicomplexa, Haemosporida) are protozoa that infect vertebrate blood cells and are transmitted by vectors. Among vertebrates, birds possess the greatest diversity of haemosporidia, historically placed in 3 genera: *Haemoproteus*, *Leucocytozoon* and *Plasmodium*, the causative agent of avian malaria. In South America, existing data on haemosporidia are spatially and temporally dispersed, so increased surveillance is needed to improve the determination and diagnosis of these parasites. During the non-breeding season in 2020 and 2021, 60 common terns (*Sterna hirundo*) were captured and bled as part of ongoing research on the population health of migratory birds on the Argentinian Atlantic coast. Blood samples and blood smears were obtained. Fifty-eight samples were screened for *Plasmodium*, *Haemoproteus* and *Leucocytozoon*, as well as for *Babesia* parasites by nested polymerase chain reaction and by microscopic examination of smears. Two positive samples for *Plasmodium* were found. The cytochrome b lineages detected in the present study are found for the first time, and are close to *Plasmodium* lineages found in other bird orders. The low prevalence (3.6%) of haemoparasites found in this research was similar to those reported for previous studies on seabirds, including Charadriiformes. Our findings provide new information about the distribution and prevalence of haemosporidian parasites from charadriiforms in the southernmost part of South America, which remains understudied.

## Graphical abstract

**Figure:**
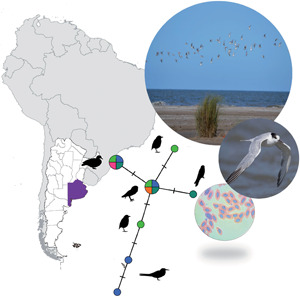

## Introduction

Wetland ecosystems harbour a wide variety of species including different parasites that often go undetected. This can negatively affect the viability of wildlife populations. The detection of parasites and pathogens is important for the identification of endemic as well as exotic diseases, in order to know the host population's health status, the pressures they face and the actions that can be developed for their conservation and management (Lebarbenchon *et al*., 2008). Wild birds are important natural reservoirs and potential dispersers of a wide variety of parasites (Kruse *et al*., 2004; Chang *et al*., 2020). In particular, migratory birds represent a basic mechanism in the emergence of new sources of infection at great distances from their original areas (or endemic areas) (Koprivnikar and Leung, 2015).

Among avian endoparasites, haemosporidians (Apicomplexa, Haemosporida) are protozoans that infect vertebrate blood cells and are transmitted by vectors (haematophagous dipterans). A great diversity of haemosporidian species were reported in birds, represented in 3 genera: *Haemoproteus* (containing 2 subgenera *Haemoproteus* and *Parahaemoproteus*), *Leucocytozoon* and *Plasmodium*, the aetiological agent of avian malaria (Bell *et al*., 2020). These parasites are globally distributed in most bird families, and the rates of infection and prevalence are variable depending on the bird order (Quillfeldt *et al*., 2011). Although it is assumed that avian haemosporidian infections are relatively benign in wild populations, the study of these parasites is important because they can affect host fitness, in some cases cause severe pathology (tissue necrosis, haemorrhages and anaemia) and usually mortality is difficult to detect (Valkiūnas, 2005; Palinauskas *et al*., 2013; Groff *et al*., 2019).

The avifauna of South America supports a high diversity of haemosporidians, whose distribution is strongly associated with its complex biogeography (Fecchio *et al*., 2019). However, most of the studies focused on passerine hosts, reflecting the need for sampling and describing avian haemosporidian parasites in non-passerine hosts (Bell *et al*., 2020).

Other blood parasites of concern in birds are piroplasmids of the genus *Babesia*, a protozoan parasite causing babesiosis, an emerging and potentially zoonotic disease transmitted by ticks. Symptoms may include anaemia, leucocytosis and depressed liver function (Ebani and Mancianti, 2021). By now, only the species *Babesia shortii* is known to cause pathogenicity in birds (Khan *et al*., 2019). Currently, studies that focus on avian *Babesia* from seabirds in South America are scarce (Quillfeldt *et al*., 2014).

The order Charadriiformes contains 19 families and 384 species (del Hoyo *et al*., 1996), most of them being seabirds and shorebirds. They have a cosmopolitan distribution and are usually associated with seas, rivers and wetlands (del Hoyo *et al*., 1996). Among Charadriiformes seabirds, representatives of the family Laridae (gulls, terns and skimmers) are widely distributed. They are mostly coastal; some are pelagic or can inhabit inland environments (Winkler *et al*., 2020). Within this family, the most studied species in the Northern Hemisphere is the common tern (*Sterna hirundo*) (Burger and Gochfeld, 1991). Common terns breed in most of Europe, Asia and North America, and undertake long-distance migratory movements to the Southern Hemisphere during the non-breeding season. They have extremely large population sizes, and are listed as Least Concern (IUCN, 2019). However, they are vulnerable to habitat deterioration and loss (del Hoyo *et al*., 1996). There is less information available about this species in its wintering grounds in the Western Hemisphere (South America). The southernmost sightings were made in Argentina (Mauco *et al*., 2001; Yorio, 2005), mostly on the Atlantic coast of Buenos Aires, which constitutes the largest roosting area of the common tern in South America (30 000 birds recorded at Punta Rasa) (Hays *et al*., 1997; Mauco *et al*., 2001).

The MalAvi database contains only 47 genetic haemosporidian lineages recovered from wild Charadriiformes (MalAvi database, accessed on 16 January 2023), and there is currently 1 record (i.e. *Leucocytozoon* sp.) in common terns (Włodarczyk *et al*., 2022). This paucity of information is probably due to the apparent absence or scarcity of blood parasites in certain groups of birds such as seabirds and shorebirds (Quillfeldt *et al*., 2010; Soares *et al*., 2016). Published information shows that of 113 seabird species studied, in only 27% haematozoan infections were found. Specifically, in the case of the family Laridae, 59% of the species studied were infected with at least 1 haemoparasitic species, mostly by *Haemoproteus* (Quillfeldt *et al*., 2011). There are some previous studies focusing on haemosporidia in charadriiforms from Argentina. In shorebirds for example, 2 studies reported negative results (D'Amico *et al*., 2007, 2008) and only 1 reported 1% of prevalence (Soares *et al*., 2016). In the case of larids, the only positives have been found in Malvinas Islands from the dolphin gull (*Larus scoresbii*) (Quillfeldt *et al*., 2010); the other study has shown negative results (Jovani *et al*., 2001).

Migratory movements can expose host populations to novel parasites as well as introduce parasites into new geographic areas (Koprivnikar and Leung, 2015). Therefore, it is of interest to explore parasitic species in migratory birds in South America, to increase their detection and to contribute to the knowledge of their diversity, with the ultimate goal of determining parasite–host distribution patterns. The aims of the present study were (i) to assess the prevalence of the Haemosporida (*Plasmodium*, *Haemoproteus* and *Leucocytozoon*) as well as *Babesia* spp. in common terns from Punta Rasa, their most important wintering site in Argentina, (ii) to identify the genetic diversity of haematozoa parasites and (iii) to compare the genetic relationships among haematozoa haplotypes in Charadriiformes from South America to those identified in previous investigations.

## Materials and methods

### Study area and sample collection

**Fig. 1.**
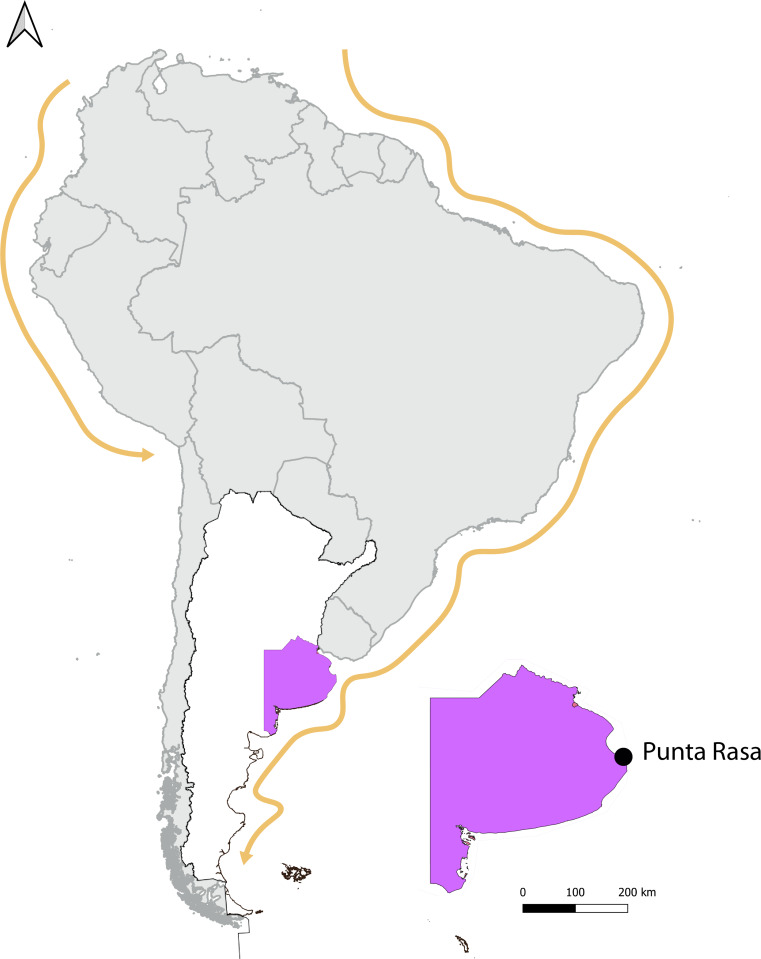
Common tern sampling location in Argentina. Arrows in orange indicate the migratory route of the species in South America.

Fieldwork was conducted during the non-breeding season between December 2020 and April 2021, in Punta Rasa, on the Atlantic coast of Argentina (Fig. 1). Punta Rasa is located in Samborombon Bay in the Río de La Plata estuary (36°17′22.8″ S; 56°46′52.9″ W) and is used by several shorebirds (e.g. sandpipers, plovers and oystercatchers) and marine birds (e.g. skimmers, terns and gulls) as roosting and feeding areas during the non-breeding season (Mauco *et al*., 2001). Birds were caught in January 2020 and February and March of 2021 using canon nets (permit number DI-2019-241). Blood samples of maximum 75 *μ*L were collected by brachial venepuncture and stored on blotting paper (Biodynamics SRL, Buenos Aires, Argentina). Blood samples on the blotting paper were stored in the dark and at room temperature in separate tubes to avoid cross-contamination until DNA isolation. For each bird, 2 thin smears on microscope slides were made. Blood films were air dried immediately in the field, and then fixed with absolute methanol and stained with Giemsa in a working solution prepared with phosphate buffer pH 7.0 (ratio 1:5) for 30 min in the laboratory.

### Blood parasite detection

#### Haemosporidians

The presence or absence of avian haemosporidians was determined through nested polymerase chain reaction (PCR) through amplification of the mitochondrial cytochrome b (cytb) gene. Total DNA was extracted using the ammonium-acetate DNA precipitation protocol (Martínez *et al*., 2009). DNA was obtained from 58 of the 60 birds sampled. NanoDrop2000c UV-Vis spectrophotometer (NanoDrop Technologies, Wilmington, USA) was used to measure the DNA concentration after extraction. If the DNA concentration was higher than 80 ng *μ*L^−1^, samples were diluted to 20 ng *μ*L^−1^. In addition, we tested the quality of the DNA by performing the PCR sexing, resulting in 97% of the samples successfully sexed. Molecular sexing was performed following the protocol of Quintana *et al*. (2008). An initial PCR was run with the primer pair HaemNFI/HaemNR3 [see Hellgren *et al*. (2004) for cycling conditions]. An aliquot of this PCR product was subsequently used as template DNA for the second PCR with specific primer pairs HaemF/HaemR2 for *Haemoproteus* and *Plasmodium* cytb gene amplification (Bensch *et al*., 2000), and HaemFL/HaemR2L for *Leucocytozoon* cytb amplification (Hellgren *et al*., 2004). The PCRs with HaemF/HaemR2 and HaemFL/HaemR2L consisted of 25 *μ*L reaction volumes containing 4 *μ*L DNA template, 1.65 *μ*L of each primer (10 mm), 12.5 *μ*L of DreamTaq Master-Mix (Thermo Fisher Scientific, Waltham, USA) and 5.2 *μ*L of nuclease-free water. Samples of different species of Columbiformes with known infections have been used as positive controls in each PCR run (Schumm *et al*., 2021). Negative controls [template replaced with double-distilled water (ddH_2_O)] were included in runs to check for possible contamination. Then, PCR products of the samples were visualized using QIAxcel Advanced (Qiagen, Hombrechtikon, Switzerland) high-resolution capillary gel electrophoresis. A positive PCR result was interpreted as an infected bird, and positive samples were Sanger sequenced bidirectionally by Microsynth-Seqlab (Sequence Laboratories Goettingen GmbH, Göttingen, Germany). For the new lineages, PCR and sequencing were performed twice to verify the results. The new cytb sequences were deposited in GenBank under the corresponding accession numbers.

To confirm the presence or absence of intracellular parasite gametocytes, smears (*n* = 120) were double-blind scanned by microscopic examination at ×1000 magnification using a light microscope (PrimoStar Zeiss, Göttingen, Germany).

#### Piroplasms

Detection of piroplasms was performed by nested PCR on the 18S rRNA gene following the protocol previously developed for the detection of *Babesia* in yellow-legged gull's (*Larus michahellis*) blood (Bonsergent *et al*., 2022). Primers 18SBp_fw and 18SBp_rev and BAB-GF2 and BAB-GR2 were used in 2 different nested PCRs to amplify the expected fragments of 1529 and 560 bp, respectively, after a primary amplification with CRYPTOF and CRYPTOR primers (Malandrin *et al*., 2010). A positive control was included in each run to ensure the PCR had worked properly. A *Babesia* infecting human, *Babesia* sp. FR1, was used in the first PCR as a PCR success control. The use of genomic DNA from a *Babesia* species not infective for birds allows the identification of eventual sample contaminations from the positive control, after sequencing. *Babesia* sp. YLG genomic DNA was used as a positive control in the second PCR. Negative controls (template replaced with ddH_2_O) were included in runs to check for possible PCR contamination.

### Phylogenetic analyses

The forward and reverse sequences of the cytb fragment were assembled and trimmed in CLC Main Workbench 7.6.4 (CLC Bio, Qiagen, Denmark). To identify haemosporidian lineages, an online BLAST (National Center for Biotechnology Information) using BLASTN 2.3.0+ (Zhang *et al*., 2000) was performed in the MalAvi database (Bensch *et al*., 2009). The resulting reference sequences were aligned with our sequences and trimmed to MalAvi sequence length (479 bp) using the program BioEdit (Hall, 1999). Sequences were considered as distinct lineages if they differ by 1 or more nucleotides. Lineages with no database records in MalAvi were considered as novel, and new MalAvi names were assigned.

Constructions of lineage networks for *Plasmodium*, using the median-joining network method were performed with PopART 1.7 (Bandelt *et al*., 1999; Leigh and Bryant, 2015) (Table 1).

**Table 1.** Taxa included in the median-joining network of mitochondrial cytb gene lineages of *Plasmodium* spp.

Lineage (MalAvi)	Host	Locality	GenBank accession no.	Reference
BT7	*Parkesia noveboracensis* (Passeriformes: Parulidae)	USA	MF817784	Smith *et al*. (2018)
FALTIN14	*Falco tinnunculus* (Falconiformes: Falconidae)	China	MT281523	Huang *et al*. (2020)
PARUS67	*Parus major* (Passeriformes: Paridae)	Sweden	KU695264	Dubiec *et al*. (2016)
SFC6	*Muscicapa striata* (Passeriformes: Muscicapidae)	Europe	DQ368389	Pérez-Tris *et al*. (2007)
TURDUS1	*Cyanistes caeruleus* (Passeriformes: Paridae)	Europe	HQ537478	Szöllősi *et al*. (2011)

## Results

Of the 58 samples, we obtained quality DNA in 56 samples. After PCR assay, we found 2 positive samples (3.6%), and there were no double infections. Only *Plasmodium* infections were detected, and no *Haemoproteus*- or *Leucocytozoon*-positive samples were found. We obtained sequences of 501 and 499 bp for the positive samples. Then sequences were assembled and edited, resulting in 479 bp fragments.

**Fig. 2.**
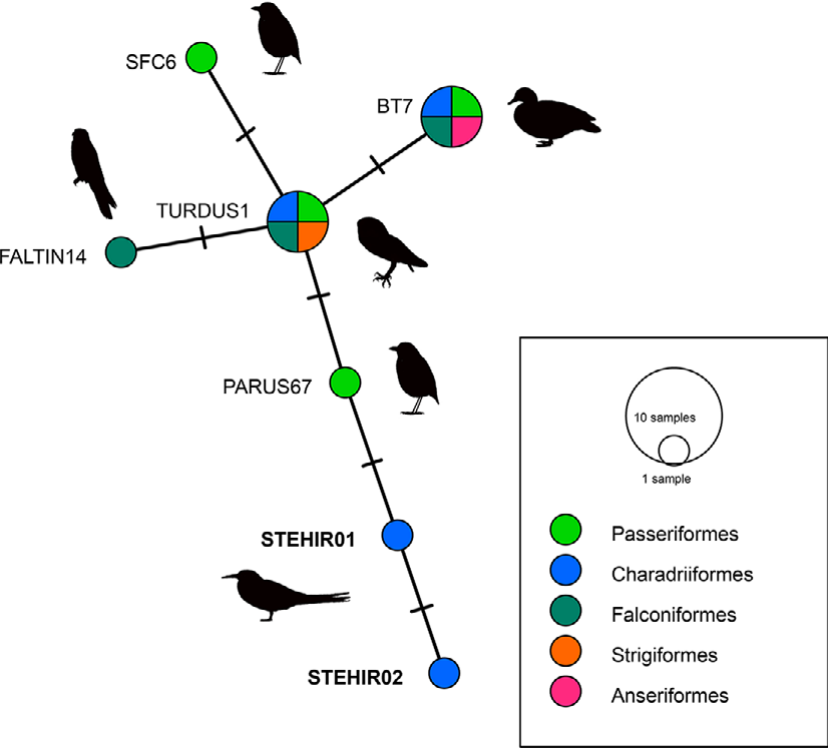
Median-joining network of mitochondrial cytb gene lineages of *Plasmodium* spp. (the 2 lineages found in the present study and the first 5 reference lineages from MalAvi, all of 479 bp, are presented in Table 1). Circles represent distinct genetic lineages, and the circle sizes are proportional to the lineage frequencies. One hatch mark represents 1 mutation. Lineage names are noted at the associated circles.

We identified 2 new *Plasmodium* lineages, named STEHIR01 (accession number ON872158) and STEHIR02 (accession number ON872159), which differ in 1 and 2 nucleotides from their closest MalAvi match PARUS67, respectively (Fig. 2). BLASTN analysis from MalAvi database revealed an identity of 99% with the *Plasmodium* lineages TURDUS1, BT7 (representing the morphospecies *Plasmodium circumflexum*), SFC6, PARUS67 and FALTIN14. The 2 new lineages appear phylogenetically clustered with *Plasmodium* lineages isolated from other bird orders such as Passeriformes, Charadriiformes, Strigiformes, Falconiformes and Anseriformes (Fig. 2). Despite positive PCR, we did not detect haemoparasites in the corresponding blood smears.

The amplification of partial 18S rRNA genes using BAB-GF2 and BAB-GR2 and 18SBp_fw and 18SBp_rev primers resulted in amplicons of non-expected sizes, most probably corresponding to non-specific primer hybridization. Sequencing and BLASTN analyses were performed on, respectively, 3 and 2 of the amplicons and confirmed non-specific amplifications. Neither *Babesia* sp. YLG nor other *Babesia*/*Theileria* piroplasms were detected in common terns' blood samples of this study.

## Discussion

Members of the Laridae family have been reported as carriers of haemoparasites in South America, such as the black skimmer (*Rynchops niger*) and the brown noddy (*Anous stolidus*) from Brazil, the swallow-tailed gull (*Creagrus furcatus*) from Galapagos, Ecuador and the dolphin gull from Malvinas Islands, Argentina (Quillfeldt *et al*., 2010, 2014; Levin *et al*., 2011; Roos *et al*., 2015). However, these lineages from Laridae are not genetically close to the new lineages found in the present study (see Supplementary information Figs S1 and S2). In the common tern there are only 2 previous studies on the subject from the Americas. One was performed at the breeding sites in the northeastern USA showing the absence of haemoparasites. This was only carried out by screening blood smears (Fiorello *et al*., 2009). The second one is a checklist of avian blood parasites from North America reporting 0% prevalence in the common tern by blood smears screening (Greiner *et al*., 1975). Recently, haemosporidians of the genus *Leucocytozoon* have been reported in common terns from Europe, but with low prevalence (<0.5%), and no *Plasmodium* positives were found (Włodarczyk *et al*., 2022).

In accordance with previous seabird studies (e.g. Fiorello *et al*., 2009; Quillfeldt *et al*., 2011; Campioni *et al*., 2018; Ilahiane *et al*., 2022; Roldán-Zurabián *et al*., 2022) haemosporidian prevalence in the common tern from the Argentinean coast was low. Avian *Plasmodium* species are distributed worldwide except in Antarctica, but only 3 species, *Plasmodium relictum*, *Plasmodium reticulum* and *Plasmodium matutinum*, have been detected in wild seabirds, the former being the most common species in birds (Quillfeldt *et al*., 2011; Włodarczyk *et al*., 2022). In our study, the new *Plasmodium* lineages differ in 1 and 2 nucleotides from their closest matching lineage PARUS67 that was found in the great tit (*Parus major*) and the marsh tit (*Parus palustris*) (Dubiec *et al*., 2016; Ellis *et al*., 2020). This can indicate a recent divergence during a host switching process. At the same time, as we were not able to observe gametocytes in the blood smears, this finding could represent an abortive malaria infection. This happens when a parasite invades a host, but cannot complete its full life cycle and be transmitted to the vector as gametocytes are absent in the blood (Palinauskas *et al*., 2016).

Other shorebirds and seabirds were analysed for haemoparasites in the Argentinean coast. For example, Jovani *et al*. (2001) analysed blood smears of 560 birds from 13 avian species, and no haemoparasites were detected. Martinez-Abrain *et al*. (2004) discussed different reasons for the apparent lack or lower presence of blood parasites in some avian species. Despite the more common explanation being the absence or scarcity of parasite vectors in some habitats, there are contradictions which show that it is not a universal explanation, and that other factors (e.g. immunological capabilities of the host, absence of the right host–parasite–vector assemblage) could be operating (Martinez-Abrain *et al*., 2004). Methodological weaknesses also could be influencing. According to our results and those from previous authors (e.g. Quillfeldt *et al*., 2010), PCR analyses are needed to avoid false negatives. This is because PCR screening techniques are reliable for detecting low intensity infections and are able to identify infections with low levels of parasitaemia that are not visible on smears (Durrant *et al*., 2006).

It is difficult to establish where birds could have been infected, since they are inter-continental migrants, and avian *Plasmodium* parasites are found worldwide. Therefore, addressing transmission in the avian malaria system turns out to be challenging. Also, in the North–South American migratory route, migratory and resident host species share the same habitats, therefore *Plasmodium* spp. lineages can switch easily between a wide range of hosts (Ricklefs *et al*., 2017).

Many species of avian *Plasmodium* use Culicidae mosquitoes belonging to different genera for completing sporogony and transmission (Valkiunas and Iezhova, 2018). Two hundred and twelve species of Culicidae mosquitoes have been recorded in Argentina. From these species, 39.75% are found in the centre of the country (Stein *et al*., 2016). This includes Buenos Aires province, where the present study was conducted. In 2010, 2 cases of lethal avian malaria were confirmed in Magellanic penguins (*Spheniscus magellanicus*). Birds were in permanent captivity in San Clemente del Tuyú (36°20′17″ S; 56°45′14″ W), the same area as the sampling site of the present study. Identifications were made by blood smears resulting in 3 species: *Plasmodium* (*Haemamoeba*) *tejerai*, *Plasmodium* (*Huffia*) sp. and *Plasmodium* (*Novyella*) sp. (Vanstreels *et al*., 2016). This indicates a chance for haemoparasitic infection at this site, but further investigations are needed in order to investigate the local transmission of avian blood parasites.

Several tick species have been proposed to transmit *Babesia* to different seabird species. They are particularly exposed to nidicolous vectors during the breeding season, given the abundance of hard or soft ticks in their colonies. In addition, it is not yet well understood whether *Babesia* species are highly host-specific (Bonsergent *et al*., 2022). Although the common terns nest on the ground, in open areas with loose substrate and with scattered vegetation, which can increase the chances of encountering ticks, we do not have sufficient information to explain the absence of *Babesia* in the common terns tested. More studies of seabirds are required to understand the parasitic dynamics and transmission ecology of *Babesia* species.

In summary, we report for the first time *Plasmodium* from wild migratory seabirds from Argentina, the first report of a *Plasmodium* in the common tern, and the second report of *Plasmodium* from wild seabirds from the temperate region of South America (Quillfeldt *et al*., 2010). The results presented here show that in the southernmost part of South America the role of wild birds (especially non-passerines) in the spread of haemoparasites remains speculative, and may change over time. In this sense, this kind of studies requires urgent attention. Increasing surveillance will allow a better understanding of parasite transmission at sites frequented by birds.

## Data Availability

The authors confirm that the data supporting the findings of this study are available within the article. Raw data are available from the corresponding and first author.

## Supplementary material

For supplementary material accompanying this paper visit https://doi.org/10.1017/S0031182023000185. click here to view supplementary material
