# Supplementary material for: Surveillance of avian malaria and related haemoparasites in common terns (Sterna hirundo) on the Atlantic coast of South America
Source: Parasitology. 2023 Mar 9;150(6):498–504. doi: 10.1017/S0031182023000185 (PMC10260293; doi:10.1017/S0031182023000185)
Supplement: Supplementary file 1 [file S0031182023000185sup001.docx]

**Supplementary material**

**Table S1.** Taxa included in the median-joining network (Figure S1) and phylogenetic tree reconstruction (Figure S2) of mitochondrial cytochrome b gene lineages of *Plasmodium* spp.

| **Lineage**  **(MalAvi)** | **Host** | **Locality** | **GenBank**  **accession no.** | **Reference** |
| --- | --- | --- | --- | --- |
| BT7 | *Parkesia noveboracensis* (Passeriformes: Parulidae) | USA | MF817784 | Smith *et al*., 2018 |
| COLPAL03 | *Columba palumbus* (Columbiformes: Columbidae) | Germany | MT888851 | Schumm *et al*., 2021 |
| CXPIP09 | *Larus argentatus* (Charadriiformes: Laridae) | Japan^*^ | - | Inumaru *et al*., 2017 |
| DENPET03 | *Rynchops niger* (Charadriiformes: Laridae) | Brazil | KJ469132 | Roos *et al*., 2015 |
| FALTIN14 | *Falco tinnunculus* (Falconiformes: Falconidae) | China | MT281523 | Huang *et al*., 2020 |
| PARUS67 | *Parus major* (Passeriformes: Paridae) | Sweden | KU695264 | Dubiec *et al*., 2016 |
| PHPAT01 | *Rynchops niger* (Charadriiformes: Laridae) | Brazil | - | Roos *et al*., 2015 |
| SFC6 | *Muscicapa striata* (Passeriformes: Muscicapidae) | Europe | DQ368389 | Pérez-Tris *et al*., 2007 |
| SGS1 | *Larus* spp. (Charadriiformes: Laridae) | Poland | - | Zagalska-Neubauer and Bensch, 2016 |
| STEHIR01 | *Sterna hirundo* (Charadriiformes: Laridae) | Argentina | ON872158 | Present study |
| STEHIR02 | *Sterna hirundo* (Charadriiformes: Laridae) | Argentina | ON872159 | Present study |
| TURDUS1 | *Cyanistes caeruleus* (Passeriformes: Paridae) | Europe | HQ537478 | Szöllősi *et al*., 2011 |

*captivity

For phylogenetic tree reconstruction, DNA sequences were aligned by CLUSTALW using MEGA version 10 (Kumar *et al*., 2018). The best-fit model of DNA sequence evolution was selected using jModeltest 2.1.7 (Darriba *et al*., 2012). According to the Akaike information criterion, we used the General Time Reversible model including invariable sites and variation among sites (GTR+I+G; Gu *et al*., 1995). Phylogenetic reconstruction was performed with BEAST 1.8.4. (Drummond *et al*., 2012). Tree priors were selected using the interface BEAUTi 1.8.4. with strict clock and a Yule speciation process (Yule, 1925; Gernhard, 2008). Markov chain Monte Carlo (MCMC) simulations were run with 25,000,000 generations and one tree was recorded every 1000 generations. In all, 10% of the trees were discarded as burn-in in TreeAnnotator (BEAST package). We validated the results of the Bayesian analyses in Tracer 1.6. (Drummond and Rambaut, 2007). The phylogenetic tree was constructed with FigTree 1.4.3 (Rambaut, 2007). Values < 0.70 are indicated by a dash.

**
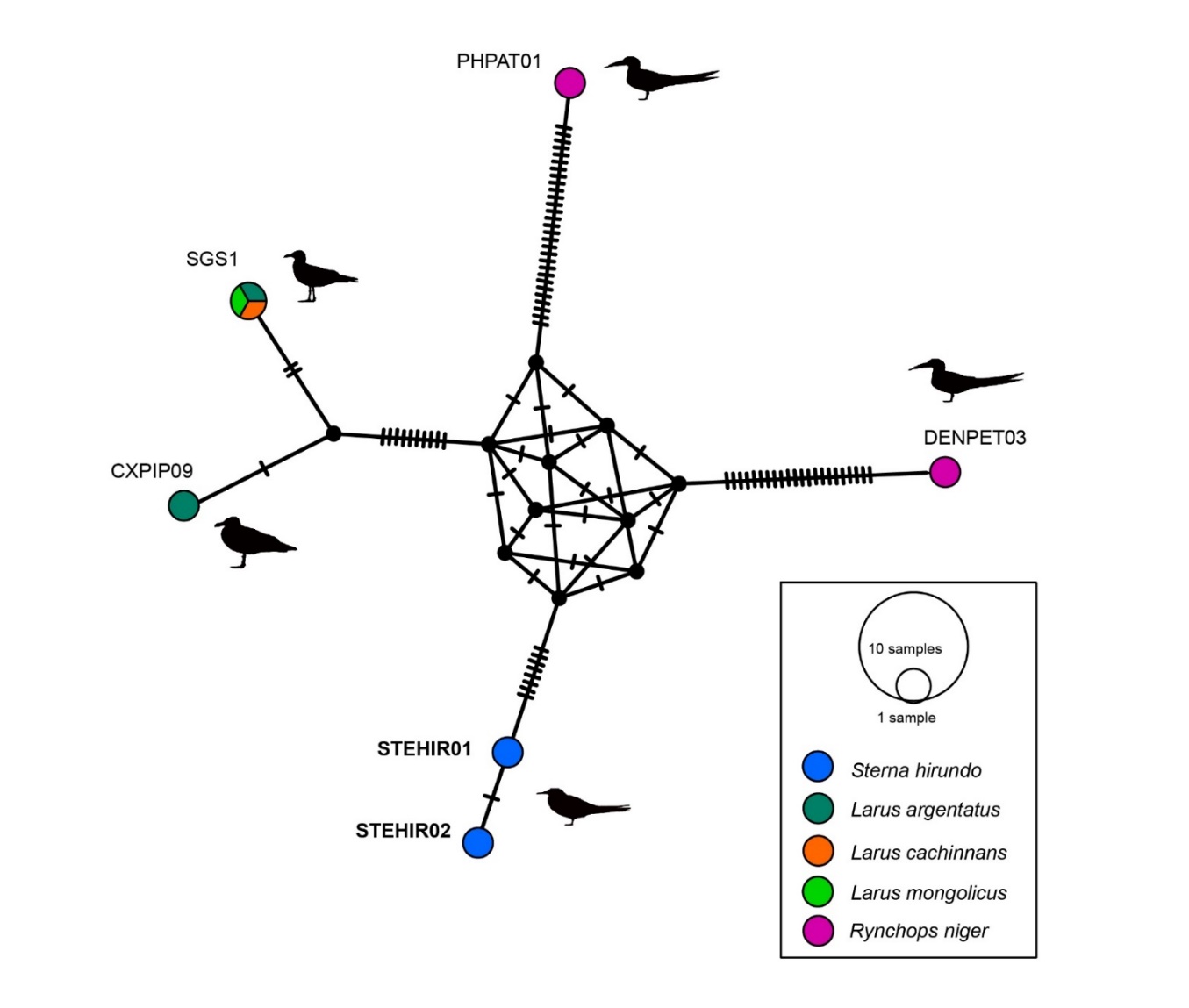
**

**Fig. S1.** Median-joining network of mitochondrial cytochrome b gene lineages of *Plasmodium* spp. (the two lineages found in the present study and reference lineages from Laridae family from wildlife present in MalAvi database, all of 479 bp, are presented). Circles represent distinct genetic lineages, and the circle sizes are proportional to the lineage frequencies. One hatch mark represents one mutation. Lineage names are noted at the associated circles

**
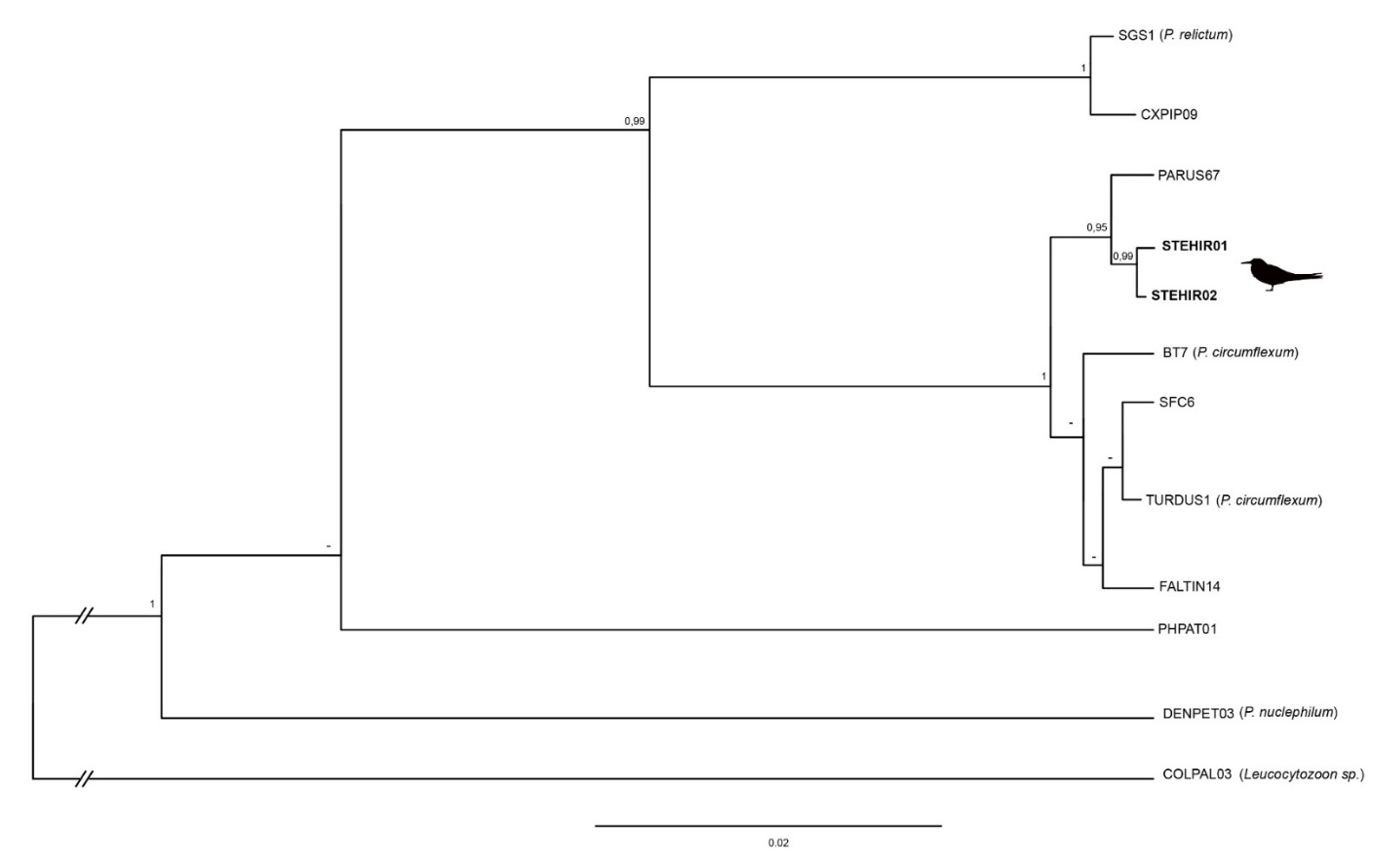
**

**Fig. S2** Phylogeny of mitochondrial cytochrome b gene lineages of *Plasmodium* spp. from Laridae family present in MalAvi database (only from wildlife) and the first five reference lineages from MalAvi blastn inferred using Bayesian analysis. Branch support values indicate Bayesian posterior probabilities. Lineages found in the present study are shown in bold. *Leucocytozoon* sp. (MT888851) was included as outgroup.

**References Supplementary material**

**Darriba, D, Taboada, GL, Doallo, R, and Posada, D (2012).** jModelTest 2: more models, new heuristics and parallel computing. *Nature methods* **9**, 772-772. https://doi.org/10.1038/nmeth.2109

**Dubiec, A, Podmokła, E, Zagalska-Neubauer, M, Drobniak, SM, Arct, A, Gustafsson, L, and Cichoń, M** (2016) Differential prevalence and diversity of haemosporidian parasites in two sympatric closely related non-migratory passerines. *Parasitology* **143**, 1320-1329. https://doi.org/10.1017/S0031182016000779

**Drummond, AJ, Rambaut, A** (2007) BEAST: Bayesian evolutionary analysis by sampling trees. *BMC Ecology and Evolution* **7**, 214. https://doi.org/10.1186/1471-2148-7-214

**Drummond, AJ, Suchard, MA, Xie, D, and Rambaut, A** (2012) Bayesian phylogenetics with BEAUti and the BEAST 1.7. *Molecular Biology and Evolution* **29**, 1969–1973. https://doi.org/10.1093/molbev/mss075

**Gu, X, Fu, YX, and Li, WH (1995)** Maximum likelihood estimation of the heterogeneity of substitution rate among nucleotide sites. *Molecular Biology and Evolution* **12**, 546–557. https://doi.org/10.1093/oxfordjournals.molbev.a040235

**Gernhard, T** (2008) Yule process. *Journal of Theoretical Biology* **253**,769–778

**Huang, X, Jönsson, J, and Bensch, S (2020).** Persistence of avian haemosporidians in the wild: a case study to illustrate seasonal infection patterns in relation to host life stages. *International Journal for Parasitology* **50**, 611-619. https://doi.org/10.1016/j.ijpara.2020.05.006

**Inumaru, M, Murata, K, and Sato, Y (2017).** Prevalence of avian haemosporidia among injured wild birds in Tokyo and environs, Japan. *International Journal for Parasitology: Parasites and Wildlife* **6**, 299-309. https://doi.org/10.1016/j.ijppaw.2017.09.007

**Kumar, S, Stecher, G, Li, M, Knyaz, C, and Tamura, K** (2018). MEGA X: molecular evolutionary genetics analysis across computing platforms. *Molecular biology and evolution* **35**, 1547. https://doi.org/10.1093/molbev/msy096

**Pérez-Tris, J, Hellgren, O, Križanauskienė, A, Waldenström, J, Secondi, J, Bonneaud, C, Fjeldså, J, Hasselquist, H, and Bensch, S** (2007) Within-Host Speciation of Malaria Parasites. *PLoS One* **2**, e235. https://doi.org/10.1371/journal.pone.0000235

**Rambaut, A** (2007) FigTree. https://tree.bio.ed.ac.uk/software/figtree/

**Roos, FL, Belo, NO, Silveira, P, and Braga, EM.** (2015). Prevalence and diversity of avian malaria parasites in migratory Black Skimmers (*Rynchops niger*, Laridae, Charadriiformes) from the Brazilian Amazon Basin. *Parasitology research* **114**, 3903-3911. https://doi.org/10.1007/s00436-015-4622-9

**Schumm, YR, Bakaloudis, D, Barboutis, C, Cecere, JG, Eraud, C, Fischer, D, Hering, J, Hillerich, K, Lormée, H, Mader, K, Masello, JF, Metzger, B, Rocha, G, Spina, F, and Quillfeldt, P** (2021). Prevalence and genetic diversity of avian haemosporidian parasites in wild bird species of the order Columbiformes. *Parasitology research* **120**, 1405-1420. https://doi.org/10.1007/s00436-021-07053-7

**Smith, JD, Gill, SA, Baker, KM, and Vonhof, MJ** (2018) Prevalence and diversity of avian Haemosporida infecting songbirds in southwest Michigan. *Parasitology Research* **117**, 471-489. https://doi.org/10.1007/s00436-017-5724-3

**Szöllősi, E, Cichoń, M, Eens, M, Hasselquist, D, Kempenaers, B, Merino, S, Nilsson, JÅ, Rosivall, B, Rytkönen, S, Török, J, Wood, MJ, and Garamszegi, LZ** (2011) Determinants of distribution and prevalence of avian malaria in blue tit populations across Europe: separating host and parasite effects. *Journal of Evolutionary Biology* **24**, 2014-2024. https://doi.org/10.1111/j.1420-9101.2011.02339.x

**Yule, GU** (1925) Yule process. *Philosophical Transactions of the Royal Society B: Biological Sciences* **213**, 21–87

**Zagalska-Neubauer, M, and Bensch, S** (2016). High prevalence of *Leucocytozoon* parasites in fresh water breeding gulls. *Journal of Ornithology*, **157**, 525-532. https://doi.org/10.1007/s10336-015-1291-5
